# Quality of Life Trajectories With Integration Into Electronic Health Records for High-Resolution Patient Outcomes: Algorithm Development and Validation Study

**DOI:** 10.2196/79834

**Published:** 2026-02-24

**Authors:** Martin Dugas, Robin Fleige, Max Christian Blumenstock, Stephan Christoph Feder, Tobias Dittrich, Niels Siegel, Celine Fabienne Bergmann, Luis Wettach, Sandra Sauer, Pavlina Lenga, Sandro M Krieg, Susanne Dugas-Breit, Lucy Joanne Kessler, Kosima Kosmalla, Fleur Fritz-Kebede, Martin Loos, Hans-Christoph Friederich, Thomas M Pausch, Matthias Ganzinger

**Affiliations:** 1 Institute of Medical Informatics Heidelberg University Hospital Heidelberg Germany; 2 Department of General Internal Medicine, Psychosomatics and Psychotherapy Heidelberg University Hospital Heidelberg Germany; 3 Department of Hematology, Oncology and Rheumatology Heidelberg University Hospital Heidelberg Germany; 4 Department of General, Visceral and Transplantation Surgery Heidelberg University Hospital Heidelberg Germany; 5 Department of Neurosurgery Heidelberg University Hospital Heidelberg Germany; 6 National Center for Tumor Diseases Heidelberg University Hospital Heidelberg Germany; 7 Department of Ophthalmology Heidelberg University Hospital Heidelberg Germany; 8 Department of Gastroenterology Heidelberg University Hospital Heidelberg Germany

**Keywords:** quality of life, electronic health records, patient-reported outcome measures, mobile devices, feasibility study

## Abstract

**Background:**

Patient-reported outcome measures (PROMs) such as health-related quality of life (HRQoL) are usually assessed at greater time intervals such as diagnostic time points, after treatment, and during follow-up. Many PROMs require frequent data collection (weekly or daily). Electronic PROMs enable high-resolution tracking but face declining response rates. Integrating PROMs into electronic health records (EHRs) could improve response rates and personalize therapy.

**Objective:**

This study aimed to evaluate the technical and clinical feasibility of high-frequency HRQoL assessments for routine care in EHRs.

**Methods:**

Patients receive emails on their mobile devices with 1-time links to a web-based app called MyEDC. This app communicates with an electronic data capture proxy in the demilitarized zone of the hospital. With a polling mechanism, these patient data are transferred to the protected hospital network and uploaded to the EHR system. HRQoL on a visual analog scale is assessed over the course of treatment in 4 clinical use cases: psychosomatics, hematology, visceral surgery, and neurosurgery.

**Results:**

Quality of life (QoL) trajectories were collected for 110 patients with daily or weekly data collection between 2 weeks and 3 months. The HRQoL analyses revealed clinically relevant findings across the 4 different medical domains. In the use case psychosomatics, 36 patients showed a significant increase in HRQoL following 4 weeks of therapy, rising from a median of 42% (IQR 32%-52%) to 60% (IQR 41%-67%; *P*=.01). An analysis of 25 patients in hematology demonstrated a significant correlation between HRQoL and 30-item QoL Questionnaire (EORTC QLQ-C30) global health status score (*P*=.02). For 26 patients in visceral surgery, a significant association was observed between HRQoL and the reported pain level (*P*<.001). The clinical feasibility was further highlighted in the neurosurgery use case, where 23 patients showed a median response time to the electronic PROM questionnaires of 5.3 (IQR 0.6-17.7) hours. HRQoL values were associated with disease-specific symptoms and scores, indicating clinical validity of this readout. Considerable variability of HRQoL was observed over time, both intraindividually and interindividually. Median area under the curve of HRQoL ranged from 0.46 to 0.79. Median time to answer ranged from 0.9 to 7.1 hours. No significant association between number of responses and age was observed.

**Conclusions:**

High-resolution QoL trajectories with EHR integration are technically and clinically feasible. They offer a novel readout beyond survival analysis or PROM end point, enabling precise disease characterization and treatment comparison.

## Introduction

At present, patient-reported outcome measures (PROMs) are typically assessed at the time of diagnosis, after initial treatment and every 3 to 6 months during follow-up [1]. However, these time intervals for PROM assessment and the length of the follow-up period are often based on clinical feasibility rather than standardized guidelines. Many established PROMs address a time interval of 1 to 2 weeks or less, for example, the 5-item World Health Organization (WHO-5) well-being index [2], 9-item Patient Health Questionnaire [3], and patient-reported outcomes-common terminology criteria for adverse events (PRO-CTCAE) [4]. Health-related quality of life (HRQoL) is a typical example of a PROM and can be assessed with instruments such as the EQ-5D [5], which addresses today’s status. Therefore, and for clinical necessity, complete PROM trajectories require more frequent data collection, depending on the instrument every 2 weeks, weekly, or even daily. Electronic collection of PROMs provides new opportunities for such frequent assessments. The time course of PROMs can be determined with this technology; for example, a recovery trajectory for cardiac surgery patients was described in the study by Mori et al [6]. However, response rate (RR) is a major issue: a review of RRs over time [7] reported an average RR for electronic PROMs of 42% at baseline, decreasing to 36% after 1 year. It is assumed that integration of digital health tools into routine care can contribute to long-term engagement and higher retention rates [8]. Current methods often rely on stand-alone apps that store PROMs in external databases, creating data silos that impede real-time access and clinician use. Integrating PROMs directly into the electronic health records (EHRs) overcomes these limitations, offering physicians a novel, comprehensive clinical readout for better-informed decisions and potentially fostering an improved patient-physician relationship.

Our hypothesis is that the integration of HRQoL from mobile devices into EHRs can improve RRs and enable the collection of HRQoL trajectories with high resolution, that is, weekly or even daily. Patients will show a higher commitment to provide HRQoL data continuously because these are sent to the medical team and thereby have a direct effect on therapy for each individual patient. From an informatics perspective, this is challenging because such data need to be collected and transferred from a large variety of mobile devices on the internet into the protected network of an EHR system. The objective of this work was to assess the technical and clinical feasibility of high-resolution HRQoL assessments, which can visualize the course of a disease from a patient perspective and are integrated with EHRs.

## Methods

### IT Architecture and Information Security

To capture QoL trajectories (QoL-T) with high resolution, that is, biweekly, weekly, or even daily, each patient used their own mobile device for data collection. The alternative would be a preinstalled device provided by the health care organization, which is associated with significant cost and logistical effort and therefore cannot provide a scalable system. Patient devices were very heterogeneous: smartphone or tablet with iOS or Android, laptop or PC, and different operating systems with different software versions. Therefore, a web-based app (HTML5) was developed for data collection, called MyEDC (my electronic data capture), which is based on OpenEDC [9,10]. This approach avoids software installations by the patient.

**Figure 1:**
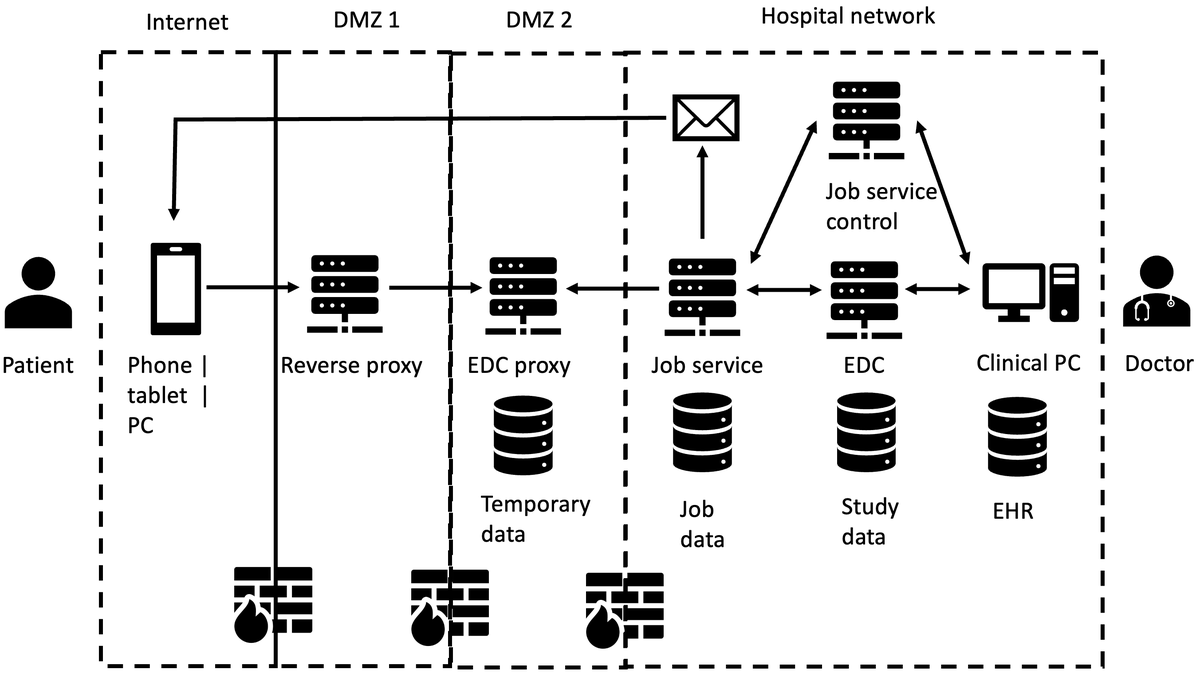
IT architecture for data transfer from patient device through hospital firewalls into internal electronic health record system. DMZ: demilitarized zone; EDC: electronic data capture; EHR: electronic health record; PC: personal computer.

Figure 1 presents the high-level IT architecture for data transfer from the patient device to the EHR. This architecture was reviewed and approved by the information security officer of Heidelberg University Hospital (UKHD). The web app was provided by an electronic data capture (EDC) proxy in the demilitarized zone, which is protected by 3 firewalls and a reverse proxy. In the internal network, a job service (programmed in Java with Spring framework) sent emails with 1-time EDC links to the patient. The job service polled data from outside to the internal network but only for registered and valid data collections. Other data packages were ignored and deleted. Valid data were transferred to an internal EDC database, which converted data into EHR format and performed an automated upload to the EHR database. A Clinical Data Interchange Standards Consortium (CDISC) operational data model [11]–compliant EDC system was used to comply with data standards in clinical trials. Owing to technical limitations of the local EHR system (i.s.h.med from Cerner Inc), files in PDF with informative document titles were generated and uploaded into the EHR.

### Short Questionnaires for High-Frequency PROM Collection

**Figure 2:**
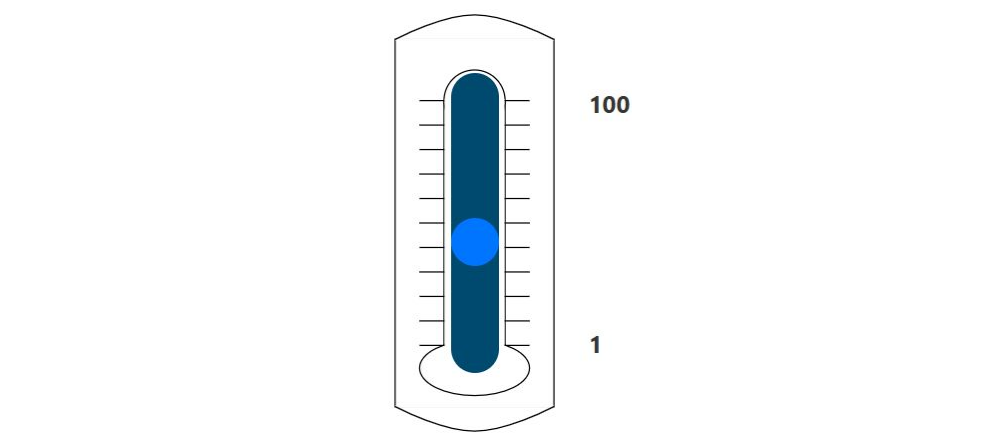
Overall health-related quality of life with a 100-level visual analog scale. General questions: How do You feel? (1=very bad, 100=very good).

Overall, HRQoL was assessed frequently using a visual analog scale (VAS) with a range from 1 to 100 (Figure 2), similar to HRQoL instruments such as EQ-5D. An HRQoL value of 0 was reserved for deceased patients. QoL-T, that is, a time series of HRQoL values, were plotted and summarized with a normalized area under the curve (AUC) per patient. Few (mostly <5) additional, disease-specific PROM items were added for each clinical use case, for example, pain in surgical patients. Simple language was used to support a wide variety of use cases, including severely ill patients and participants with limited language skills. These questionnaires were designed by clinical experts to assess patient trajectories regarding effectiveness and side effects of routine therapy.

### Statistical Analysis

Statistical analyses were performed using R (version 4.4.0). Differences between paired observations were assessed using the Wilcoxon signed-rank test. Associations between continuous or ordinal variables were investigated using the Spearman rank correlation coefficient. For all tests, a 2-sided *P* value threshold of .05 was considered statistically significant. AUC was calculated with the trapezoidal rule using the mean of adjacent points, normalized by total time, and scaled to a value range from 0 to 1. A 95% confidence band for average QoL was calculated as a point-wise 95% CI of the mean. The participation rate was defined as the percentage of eligible patients who consented to participate in the study out of the total number of individuals invited. To assess longitudinal data quality, we calculated the RR at each assessment time point, defined as the percentage of participating patients who completed the questionnaire at that specific interval. Time to answer was calculated for completed questionnaires. Clinical feasibility for QoL-T was assessed regarding completeness with the median RR across all time points. Timeliness of patient answers to short PROM questionnaires was described with median time to answer.

### Ethical Considerations

This study was conducted in accordance with the ethical guidelines of the Declaration of Helsinki. The research was approved by the ethics committee of the Medical Faculty of Heidelberg University (S-607/2023 and S-255/2025). The privacy and confidentiality of participants were maintained by ensuring that all study data were deidentified. Informed consent was obtained from all participants; this included the explicit right to opt out or withdraw from the study at any time. No direct compensation was provided for participation.

### Clinical Use Cases

#### Overview

Four different use cases were selected to assess the clinical feasibility of QoL-T with EHR integration in psychosocial and somatic medicine. PROMs are clinically relevant across these distinct disciplines because they capture the patient’s subjective experience of disease burden, treatment efficacy, and functional recovery. PROMs can provide insights into mental well-being (psychosomatics), treatment toxicity and fatigue (hematology), postsurgical functional status (visceral surgery), and pain (neurosurgery). Overall, 110 out of 184 (59.7%) patients agreed to participate.

#### Psychosomatics

As a typical example for psychosocial medicine, 36 inpatients with anorexia nervosa (Department of General Internal Medicine and Psychosomatics, UKHD) used the system with their own devices (predominantly smartphones) between August 2024 and April 2025. Three additional patients did not participate because they did not have a suitable device or did not want to use it for this purpose. Data were collected once a week for at least a month per patient. In addition to HRQoL-VAS, further widely used psychometric instruments to assess depression and anxiety (4-item Patient Health Questionnaire, PHQ-4 [12]), somatic symptoms (12-item Somatic System Disorder, SSD-12 [13]), short symptom checklist (Symptom-Checkliste Kurzversion, SCLK-11 [14]), work ability (Work Ability Index [15]), reflective functioning (Reflective Functioning Questionnaire, RFQ [16]), and 8-item Eating Disorder Examination-Questionnaire (EDE-Q8) [17] were applied. EDE-Q8 is an established clinical score to track symptoms of anorexia nervosa.

#### Hematology

A total of 25 outpatients with myeloma (Department of Hematology, Oncology, and Rheumatology, UKHD) used the system with their own devices between September 2024 and March 2025. In addition, 9 patients did not participate (technical reasons: 6, overwhelmed by medical situation: 2, and other: 1). Data were collected once a day for a period of 2 weeks during chemotherapy. In addition to HRQoL-VAS, side effects were collected according to PRO-CTCAE [18].

#### Visceral Surgery

In total, 26 inpatients (Department of General, Visceral, and Transplantation Surgery, UKHD) used the system with their own devices between January 2025 and April 2025; 59 screened patients did not participate (technical reasons: 15, overwhelmed by medical situation: 7, data protection concerns: 12, language barrier: 10, and other: 15). Patients with pancreatic diseases requiring surgical therapy were recruited for data collection at admission the day before surgery. Data were collected once a day for a period of 2 weeks at baseline before and immediately after the surgical procedure. In addition to HRQoL-VAS, the following items were collected: postoperative pain, nausea, feverish feeling, bowel movements, and the ability to drink or eat.

#### Neurosurgery

A total of 23 inpatients with spinal stenosis or herniated disc (Department of Neurosurgery, UKHD) used the system on their own devices between March 2025 and April 2025. Data were collected once a day for a period of 2 weeks immediately after the surgical procedure. In addition to HRQoL-VAS, the following items were collected: pain at the surgical site, leg pain, arm pain, and dysesthesia. Additionally, 9 patients did not use MyEDC (technical assistance needed: 6, language barrier: 1, overwhelmed by medical situation: 1, and other: 1).

## Results

Patients entered HRQoL data via 1-time EDC links as described in the Methods section. The first question addressed overall QoL with a 100-level VAS. Different variants of this VAS were used. In visceral surgery, a vertical VAS was used (Figure 2); in psychosomatics and neurosurgery, a horizontal VAS was applied; and in hematology, a vertical VAS with an inverted scale (distress 1-100) was provided. In addition to the VAS, a set of disease-specific questions (such as pain for surgical patients) were asked. Data transfer from outside to the EHR system was implemented using a polling mechanism. On the basis of these data, EHR documents were generated automatically as a manual document list view (Figure S1 in Multimedia Appendix 1) and a detailed document view (Figure S2 in Multimedia Appendix 1), similar to a laboratory report. In a 9-month period (August 2024-April 2025), a total of 110 patients from UKHD participated in 4 clinical use cases.

**Figure 3:**
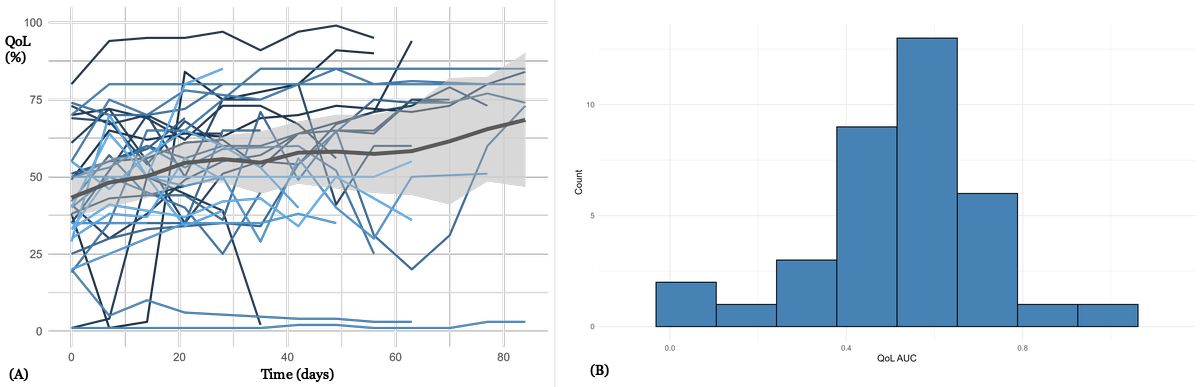
(A) Health-related quality of life trajectories for 36 patients from psychosomatic medicine. Each line corresponds to a patient. A 95% confidence band is provided. (B) A histogram of the quality of life per patient (standardized area under the curve for each patient; median 0.55, IQR 0.43-0.64). QoL AUC: quality of life area under the curve.

Figure 3 presents QoL-T for 36 patients from psychosomatic medicine (32 female and 4 male patients; median age 27, IQR 18-58 years), who received weekly emails to collect HRQoL values. In the beginning, there was a wide range of HRQoL values. Patients provided HRQoL data with a relatively high level of completeness over the treatment period (median RR 92%, IQR 86%-97%in the first month). The participation rate was 92% (33/36; ie, highest of all 4 use cases). On average, HRQoL values improved over time, indicating a positive effect of therapy (median HRQoL at baseline 42%, IQR 32%-52%, median HRQoL after 4 weeks 60%, IQR 41%-67%; *P*=.01). There is a negative correlation (significance level was not reached) between HRQoL and EDE-Q8 (Spearman correlation=–0.123, *P*=.06; Figure S3 in Multimedia Appendix 1), which indicates that decreased QoL is associated with higher levels of eating disorder symptoms.

**Figure 4:**
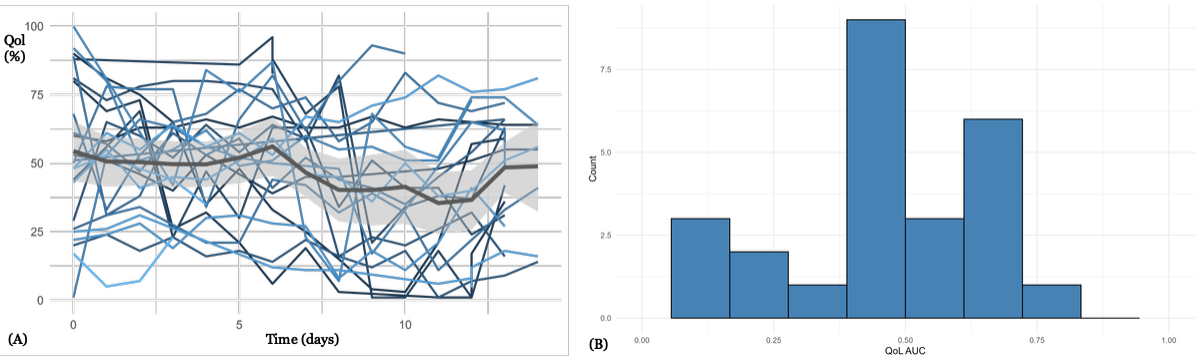
(A) Health-related quality of life trajectories for 25 patients from hematology. Day 0 corresponds to start of chemotherapy. (B) Histogram of quality of life per patient (standardized area under the curve; median 0.46, IQR 0.41-0.62). QoL AUC: quality of life area under the curve.

Figure 4 presents QoL-T for 25 patients from hematology (14 female and 11 male patients; median age 63, IQR 45-71 years), who received daily emails to collect HRQoL. Patients provided HRQoL data with a high level of completeness over the treatment period (median RR within 1 week 83%, IQR 80%-88%). There is a significant correlation of HRQoL and 30-item QoL Questionnaire (EORTC QLQ-C30) global health score (Spearman correlation=0.498, *P*=.02; Figure S4 in Multimedia Appendix 1). On average, HRQoL values deteriorate over a time interval of approximately 12 days, indicating the side effects of intensive chemotherapy.

**Figure 5:**
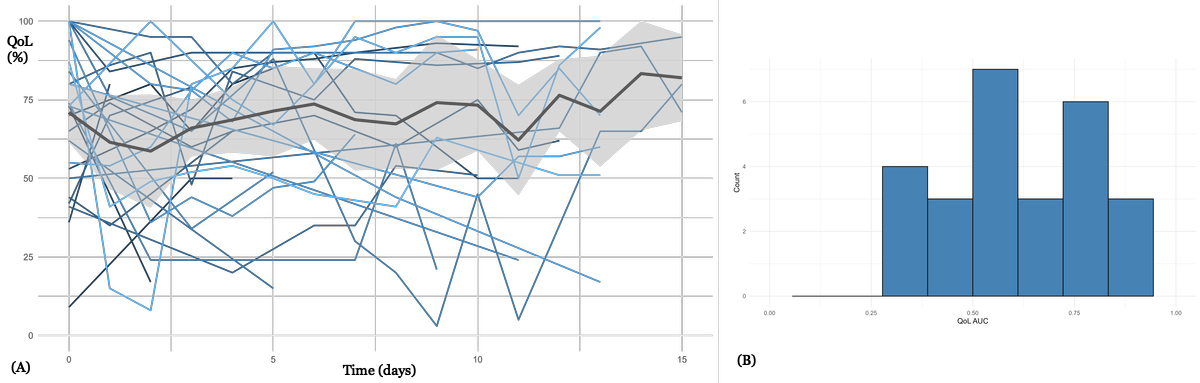
(A) Health-related quality of life trajectories for 26 patients from visceral surgery. Day 0 corresponds to the day before surgery. (B) Histogram of quality of life per patient (standardized area under the curve; median 0.59, IQR 0.49-0.77). QoL AUC: quality of life area under the curve.

Figure 5 presents QoL-T for 26 patients from pancreatic surgery (10 female and 16 male patients; median age 66, IQR 42-83 years), who received daily messages on the day before surgery and 2 weeks after the surgical intervention. Participants achieved a median RR in the first week of 46%, IQR 46%-58%. The participation rate was 31% (8/26; ie, lowest of all 4 use cases). There is a highly significant association between HRQoL and pain levels (Spearman correlation=−0.552, *P*<.001; Figure S5 in Multimedia Appendix 1), which indicates that pain is associated with decreased QoL.

**Figure 6:**
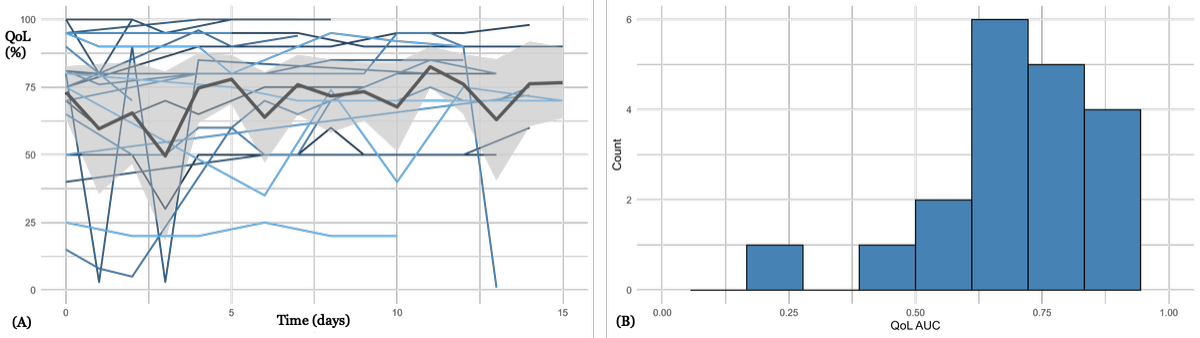
(A) Health-related quality of life trajectories for 23 patients from neurosurgery. Day 0 corresponds to the day before surgery. (B) Histogram of quality of life per patient (standardized area under the curve; median 0.79, IQR 0.62-0.90). QoL AUC: quality of life area under the curve.

Figure 6 shows QoL-T for 23 patients from neurosurgery (7 female and 16 male patients; median age 64, IQR 21-85 years), who received daily messages 2 weeks after the surgical intervention. Participants achieved a median RR in the first week of 43%, IQR 31%-61%. A negative correlation between HRQoL and pain level was observed, which aligns with the direction of the association seen in visceral surgery. However, this did not reach statistical significance (Figure S6 in Multimedia Appendix 1).

QoL-T in Figures 3-6 show considerable variability, both intraindividually (over time) and interindividually (comparison between patients). There are interpretable patterns; for example, in Figure 3 there is an average improvement of HRQoL during the course of inpatient treatment, or in Figure 4, there is an HRQoL change during the course of chemotherapy.

We did not find a significant effect of patient sex on response behavior (for details, see Figures S7-S10 in Multimedia Appendix 1). Of note, we did not observe a significant association between patient age and response count in any use case (for details, see Figures S11-S14 in Multimedia Appendix 1). We assessed time from email notification to patient answer in all use cases. The lowest time to patient answer was observed in psychosomatics (median 0.9 hours, IQR 0.3-8.0; Figure S15 in Multimedia Appendix 1). In hematology, median time to answer was 6.7 hours, IQR 1.5-17.2 (Figure S16 in Multimedia Appendix 1); in visceral surgery, median time to answer was 7.1 hours, IQR 2.9-18.9 (Figure S17 in Multimedia Appendix 1), and in neurosurgery, median time to answer was 5.3 hours, IQR 0.6-17.7 (Figure S18 in Multimedia Appendix 1).

## Discussion

### Overview

At present, survival (overall or progression free) is the most established primary end point in clinical research, especially in oncology [19]. Survival has the advantage that it can be collected with high precision in time (day of death) and completeness. However, survival is a crude measure for therapeutic success because it ignores PROMs such as QoL. Therefore, PROMs are increasingly used as end points in clinical studies [20]. This approach also has limitations because it only assesses the outcome at the end of an observation interval but not the patient trajectory: when 2 therapies result in the same average HRQoL after 1 year, therapy A can still be superior to therapy B, if HRQoL is significantly better during the time course of this year. Many established HRQoL instruments such as the WHO-5 well-being index, Functional Assessment of Cancer Therapy-General (FACT-G), or EQ-5D have short recall periods (<2 weeks). Consequently, frequent data collection (every 2 weeks up to daily) is needed for complete, high-resolution HRQoL trajectories. This is a methodological challenge because low RRs were reported in several settings.

### Principal Findings

Our hypothesis was that assessing HRQoL on patient’ mobile devices and integrating these findings into the EHR system can enable such high-resolution collection of HRQoL because a patient would be motivated to contribute to their own therapy. In our setting, a patient could directly inform their treatment team about QoL and symptoms. We could show that this approach is actually working in 4 completely different medical domains (psychosomatics, hematology, visceral surgery, and neurosurgery), both from a technical and a clinical perspective. Clinical staff emphasized the value of EHR integration because it supports patient care, for example, regarding pain management. Our proposed IT architecture proved itself in pilot operation. Patients were responding to these HRQoL requests on a regular basis.

What to measure regarding patient outcomes, including the frequency of assessment, was described as a key challenge [8]. We applied a 100-level VAS with a question in simple language because, from a clinical point of view, long texts are not suitable for seriously ill patients (eg, intensive care setting). It was demonstrated that even for those patients (eg, directly after major surgery), a large proportion is able and willing to answer HRQoL questions with their smartphones on a daily basis. In the European Union, software that calculates a clinical score may be classified as a medical device, depending on its intended use and clinical impact. While a single-item HRQoL question may be viewed as “clinical documentation,” any software-based assessment intended to influence diagnostic or therapeutic decisions requires rigorous validation. Given the vast technical heterogeneity of patient-owned smartphones, the technical and clinical validation required for such software to meet medical device standards remains a significant hurdle for large-scale implementation. More complex HRQoL instruments, such as the EORTC QLQ-C30, can provide a more detailed assessment of different QoL dimensions but require substantially more documentation effort. We propose to use such instruments in larger time intervals combined with high-frequency short HRQoL assessments. In our study, patients of almost all age groups were capable to do this data collection. The clinical team or family members can support patients who are not able to handle a mobile device.

The median time from email to answer was <8 hours; therefore, daily or weekly assessments are feasible. We propose starting the VAS with 1 and assigning an HRQoL value of 0 only to patients who are deceased. By this means, HRQoL values can be directly transformed into survival data (dead=HRQoL 0 and alive=HRQoL 1-100). This enables a direct comparison of HRQoL trajectories with survival, which is the current standard end point.

### Comparison With Prior Work

PROMs integrated with EHR were described previously [21]. High-resolution HRQoL trajectories within the EHR constitute a novel readout for the course of disease. This level of detail was previously unavailable and, to our knowledge, has not been comprehensively established for any disease. We demonstrated that this approach is feasible across multiple, clinically distinct domains, ranging from psychosomatics to visceral surgery. HRQoL trajectories could support early detection of complications. In contrast to survival analysis, effects of interventions such as chemotherapy or surgery on QoL can be assessed quantitatively with HRQoL trajectories. These trajectories can potentially enable sensitive comparison of different medical interventions in the future and change the course of therapy.

A related method is ecological momentary assessment [22], which captures real-time data about patients’ experiences and behaviors in their natural environment. A similar approach such as in our pilot study was reported with the eRAPID system [23], which was applied for patients with breast, gynecological, and colorectal cancer. With eRAPID, the patient logged onto a web-based system (Qtool) to complete online questionnaires. To simplify this step, we sent a 1-time EDC link to the patient (ie, patient does not need an individual password). We used a CDISC-compliant EDC database to make the approach more scalable: the Medical Data Models portal [24] provides ≥25.000 questionnaires for download, which can be used for data collection with CDISC-compliant systems. Of note, the eRAPID team from Leeds was able to demonstrate that PROM collection as intervention can improve patient outcomes in patients with tumor (physical well-being and self-efficacy) [25]. Improved patient outcomes induced by PROM collection was also reported in other medical domains such as orthopedics [26]. In the field of cardiology, it was reported that a disease-specific PROM score is the strongest predictor for survival of heart failure [27].

### Limitations and Future Work

Our findings are based on a single site pilot study with a relatively small sample size for each clinical use case. Consequently, our analysis may have lacked sufficient statistical power to detect smaller effect sizes. Due to technical constraints of the local EHR system, integration was limited to PDF files. Fast Healthcare Interoperability Resources–based EHR integration can enable improved support for clinical workflows. Further research is needed to define the best time intervals and most suitable assessment instruments for HRQoL collection. Most probably, these parameters are disease-specific. In some use cases, HRQoL measurements can be extended to the period before therapy. The rate of patients who accepted the electronic system varied considerably between use cases: in psychosomatics, >90% agreed to participate. In contrast, in visceral surgery, only about one-third of patients were willing or able to collect HRQoL data on the smartphone in the perioperative phase. HRQoL has several dimensions (eg, physical and psychosocial QoL). To compare 2 treatment schemes regarding HRQoL, a reduction to 1 dimension is needed (is therapy A better than B?). Therefore, further research is needed to define the most appropriate calculation of overall HRQoL. Another research topic is the metric to aggregate a series of HRQoL values. In this pilot study, we proposed AUC of HRQoL over time. Patient questionnaires can have a significant nonresponse bias, for example, language proficiency has a significant effect on RR [28]. In our setting, some patients had no access to a smartphone. Therefore, additional data capture methods (eg, tablet and interview by a health care professional) are needed for complete HRQoL data. At present, smartphones are used by >95% of the population in high-income countries [29]. Electronic HRQoL collection software needs to be further improved, given the disease-related limitations for patients compared with the general population. Software with high usability can contribute to address this problem: for instance, the study by Carter et al [30] reported an increase in PROM completeness from 24.9% to 67.0% by user-centered software design. Future work will focus on leveraging HRQoL trajectories to provide clinical decision support and enabling physicians to personalize therapeutic interventions, while simultaneously establishing a sensitive, dynamic outcome measure for future clinical trials.

### Conclusions

High-resolution QoL-T with EHR integration are clinically feasible in a broad range of clinical settings. Collection of HRQoL data via patients’ mobile devices and secure data transfer to the protected EHR is technically feasible. These trajectories can provide a novel readout, which is not covered by survival analysis or a PROM end point. These data can potentially be useful to characterize diseases and compare different therapies with high sensitivity and time resolution.

## Appendix

Multimedia Appendix 1 Data regarding electronic health record integration, correlation analyses, and time to patient answers.

Multimedia Appendix 2 Program code for quality of life trajectories (trajectory.R).

Multimedia Appendix 3 Example data for quality of life trajectories (example.R).

## Abbreviations

AUC: area under the curve
CDISC: Clinical Data Interchange Standards Consortium
EDC: electronic data capture
EHR: electronic health record
EDE-Q8: 8-item Eating Disorder Examination-Questionnaire
EORTC QLQ-C30: 30-item Core QoL Questionnaire
FACT-G: Functional Assessment of Cancer Therapy-General
HRQoL: health-related quality of life
PHQ-4: 4-item Patient Health Questionnaire
PRO-CTCAE: patient-reported outcomes–common terminology criteria for adverse events
PROM: patient-reported outcome measure
QoL: quality of life
QoL-T: quality of life trajectories
RFQ: Reflective Functioning Questionnaire
RR: response rate
SCLK-11: Symptom-Checkliste Kurzversion
SSD-12: 12-item Somatic System Disorder
UKHD: Heidelberg University Hospital (Universitätsklinikum Heidelberg)
VAS: visual analog scale
WHO-5: 5-item World Health Organization well-being index

## Data Availability

Anonymized data and data analysis software for this manuscript are available in Multimedia Appendices 2 and 3. Access to individual patient data can be requested from the Medical Data Integration Center of Heidelberg University Hospital. Data access requests will be reviewed by the Use and Access Committee of Heidelberg University Hospital.
