# Supplementary material for: Quality of Life Trajectories With Integration Into Electronic Health Records for High-Resolution Patient Outcomes: Algorithm Development and Validation Study
Source: J Med Internet Res. 2026 Feb 24;28:e79834. doi: 10.2196/79834 (PMC12976594; doi:10.2196/79834)
Supplement: Multimedia Appendix 1 [file jmir_v28i1e79834_app1.docx]

Supplement

## EHR integration (Visceral Surgery Patients)


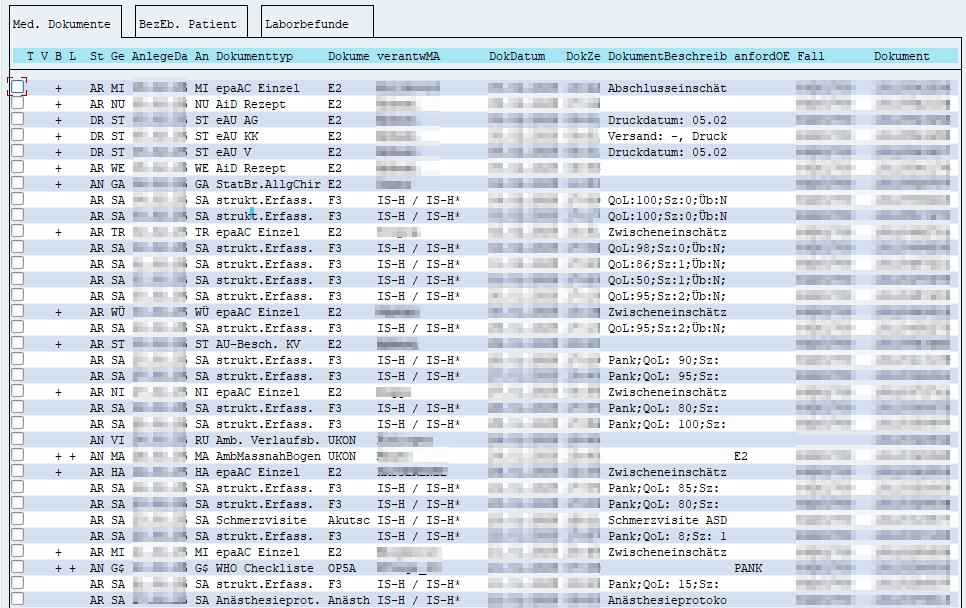


Fig. S1: Document list for a patient in the EHR system of Heidelberg University Hospital (i.s.h.med). Each line corresponds to one document. In column "DokumentBeschreib" (=document description) a customized document title is provided, e.g. "QoL:100" for quality of life 100 and "Sz:0" for "Schmerz" (=pain) 0.


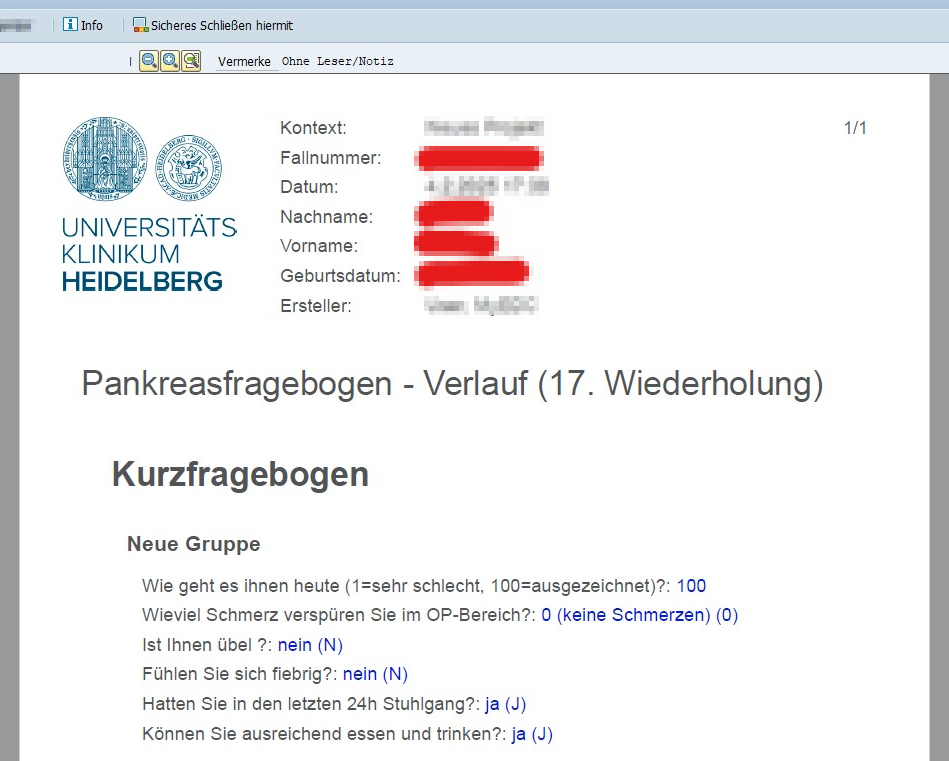


Fig. S2: Example for an EHR document (in German), which was generated automatically from data entry on the smartphone by the patient. He/she reports a quality of life level 100, pain level 0, no nausea, no fever, bowel movement in the past 24 hours, and ability to eat and drink.

## Correlation HRQoL - EDE-Q8 (psychosomatics)


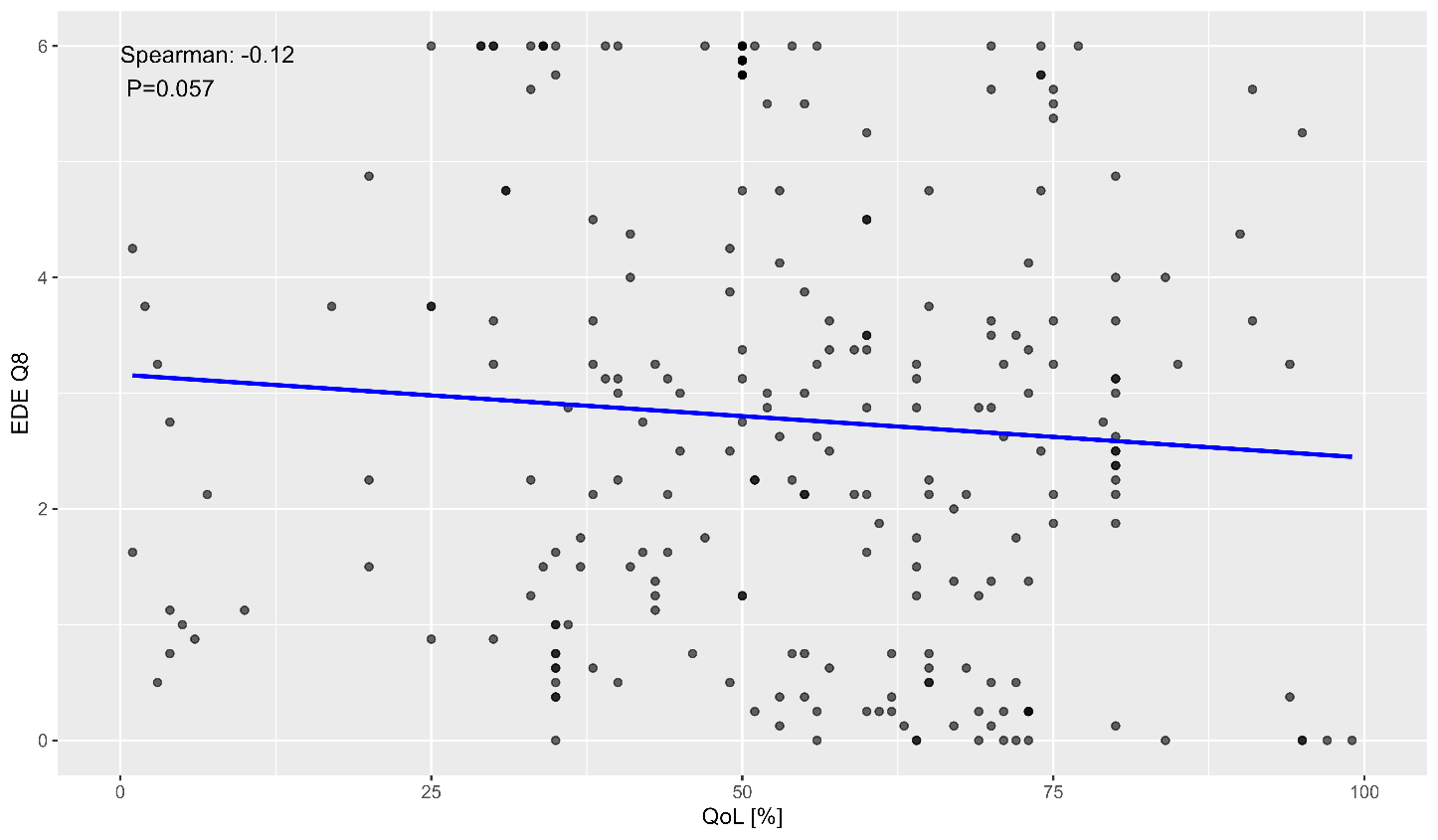


Fig. S3: Scatter plot of Eating Disorder Examination Questionnaire 8 (EDE-Q8) level and quality of life (use case psychosomatics). A high EDE-Q8 level is associated with low HRQoL.

## Correlation HRQoL - QLQ C30 Global Health (hematoloy)


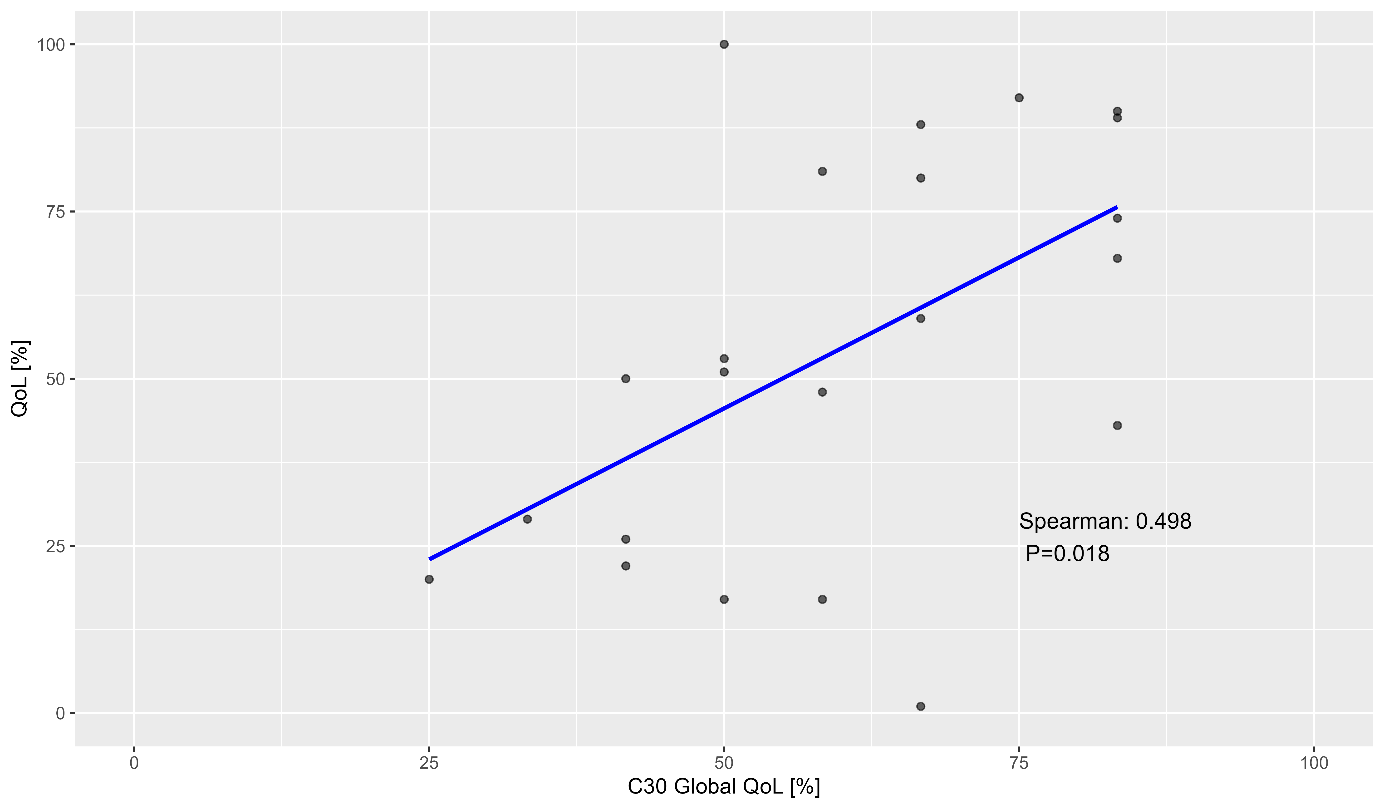


Fig. S4: Scatter plot of QLQ C30 global health level and quality of life. A high C30 global health score is associated with high HRQoL.

## Correlation HRQoL - Pain (visceral surgery)


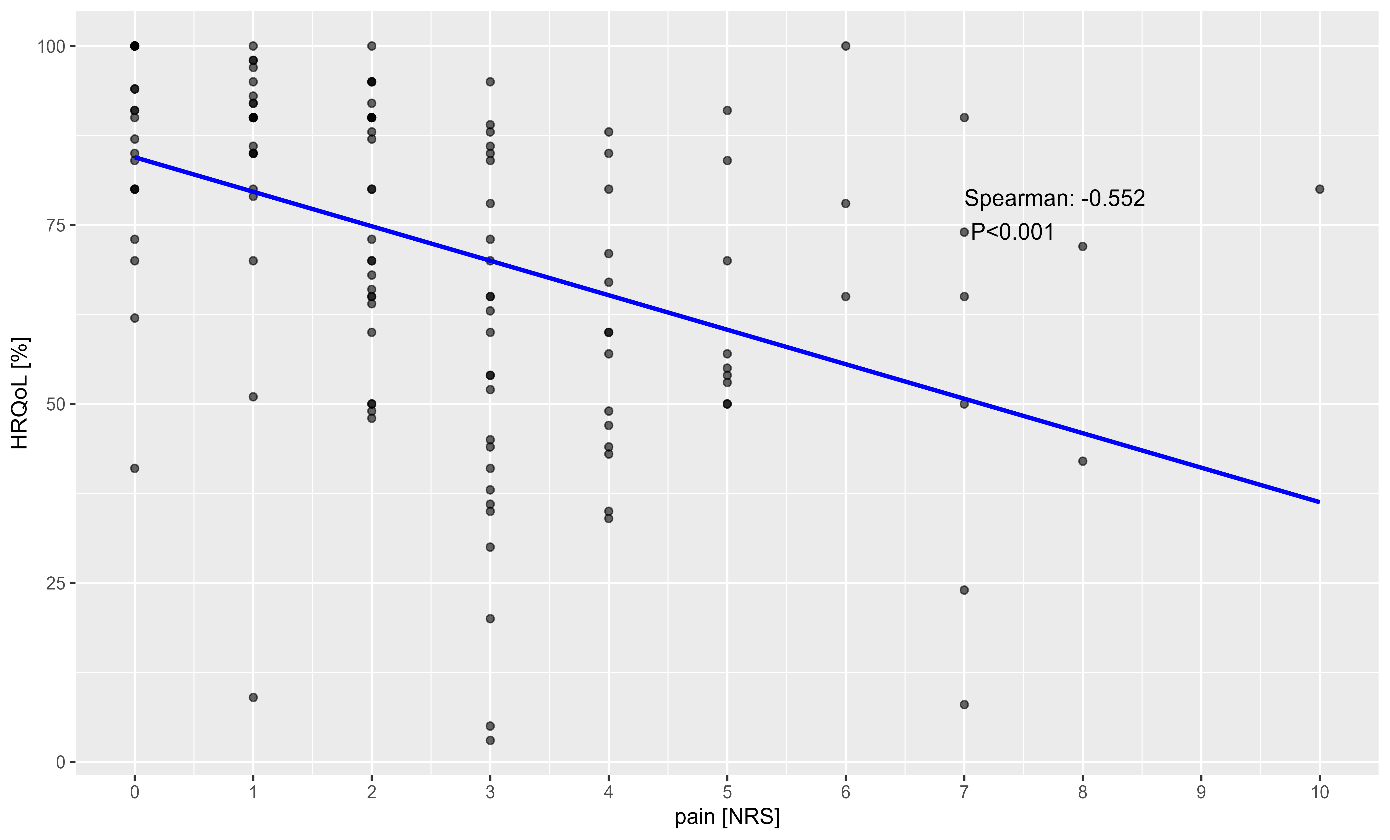


Fig. S5: Scatter plot of pain level and quality of life. High pain level is significantly associated with lower HRQoL.

## Correlation HRQoL - Pain (neurosurgery)


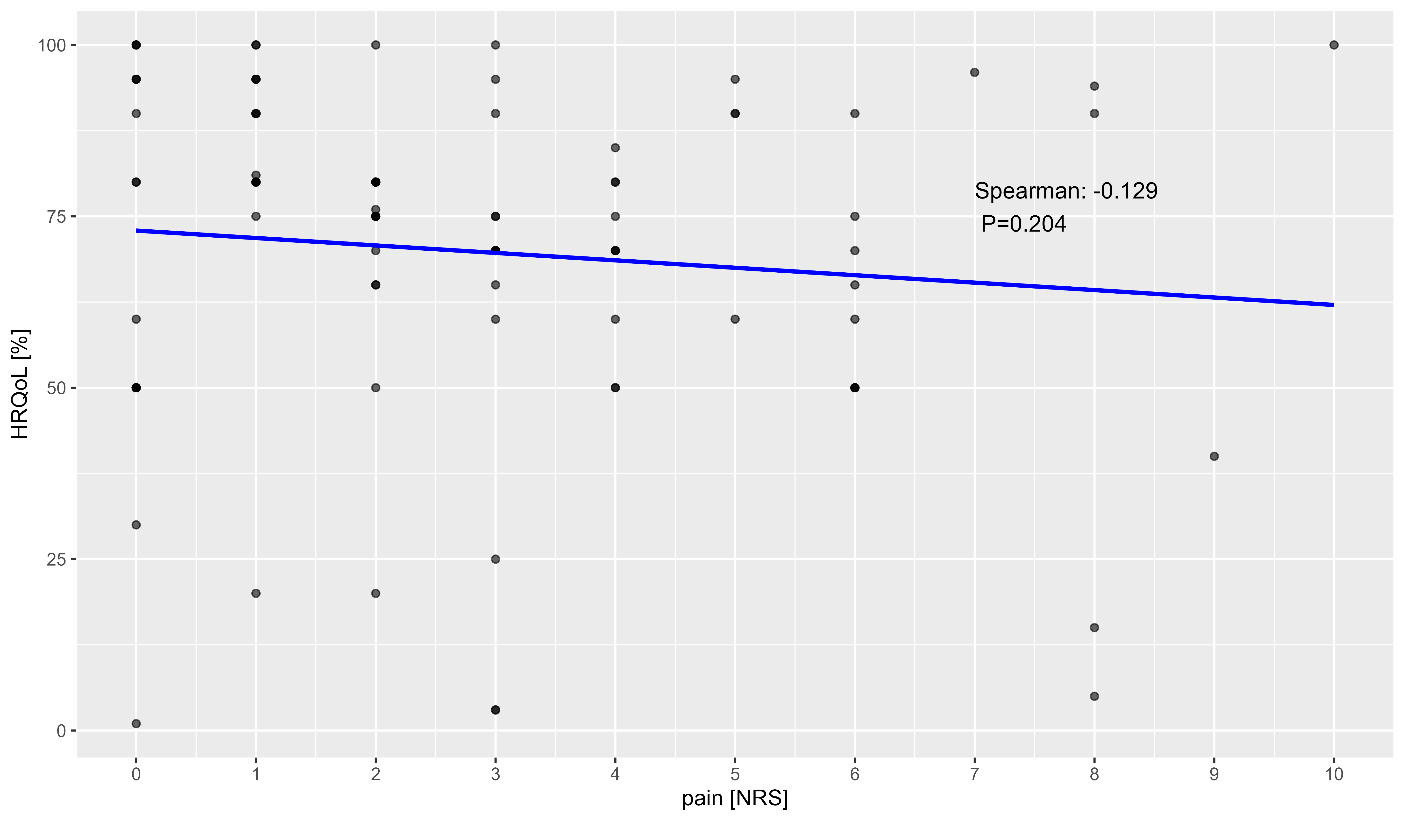


Fig. S6: Scatter plot of pain level and quality of life. High pain level is associated with lower HRQoL.

## Responses by sex


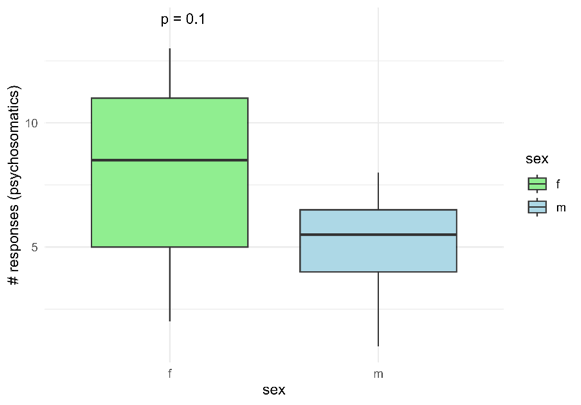


Fig. S7: Boxplots of responses per patient for females and males (use case psychosomatics).


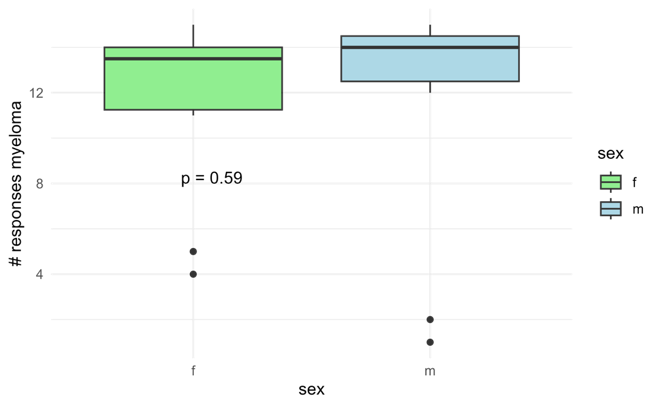


Fig. S8: Boxplots of responses per patient for females and males (use case hematology).


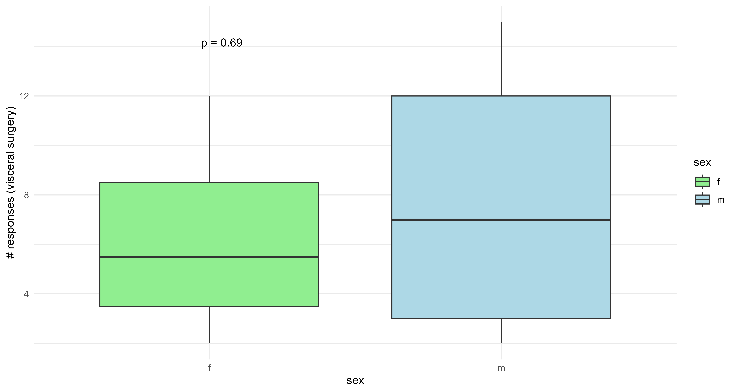


Fig. S9: Boxplots of responses per patient for females and males (use case visceral surgery).


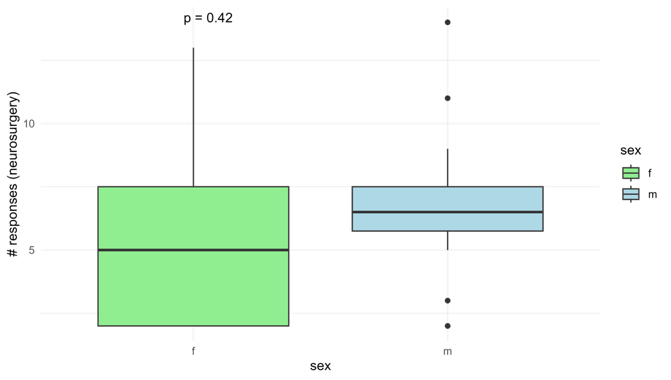


Fig. S10: Boxplots of responses per patient for females and males (use case neurosurgery).

## Responses by age


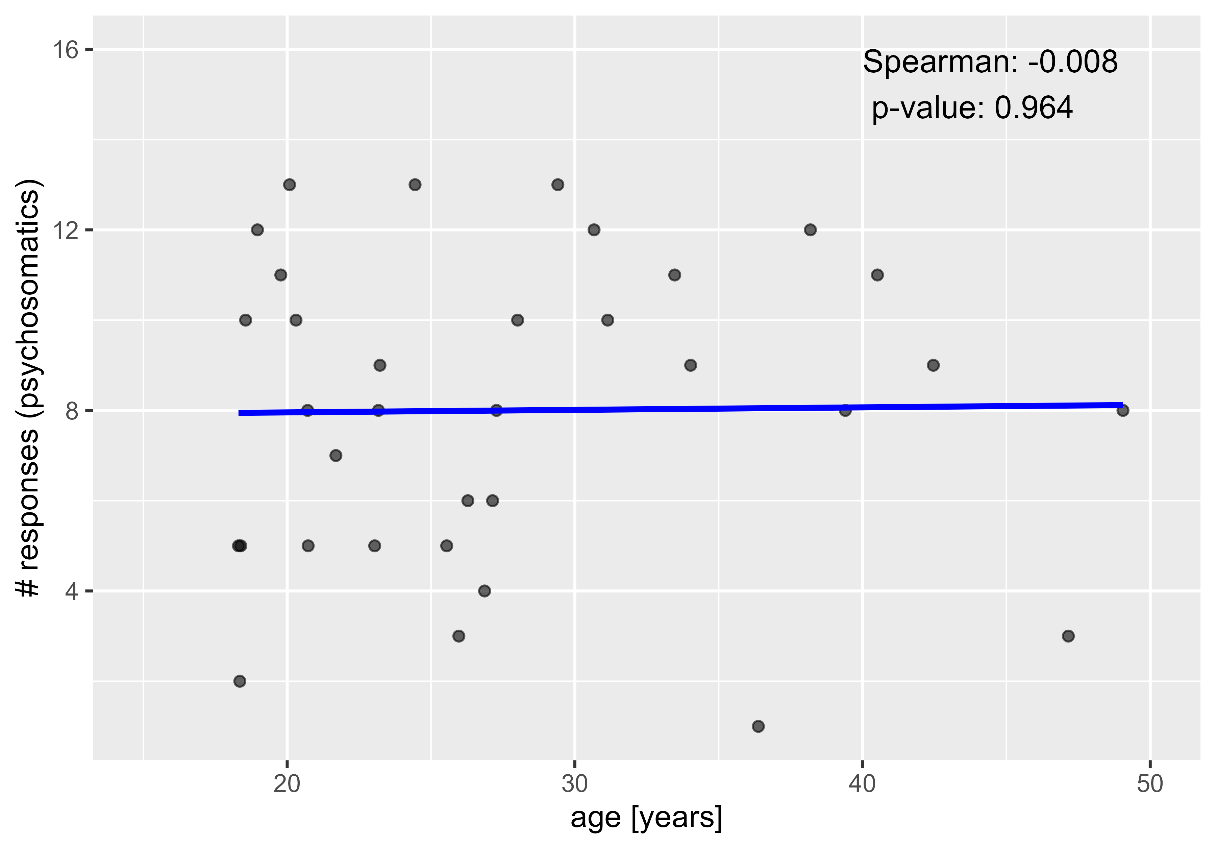


Fig. S11: Scatterplot of responses per patient by age (use case psychosomatics).


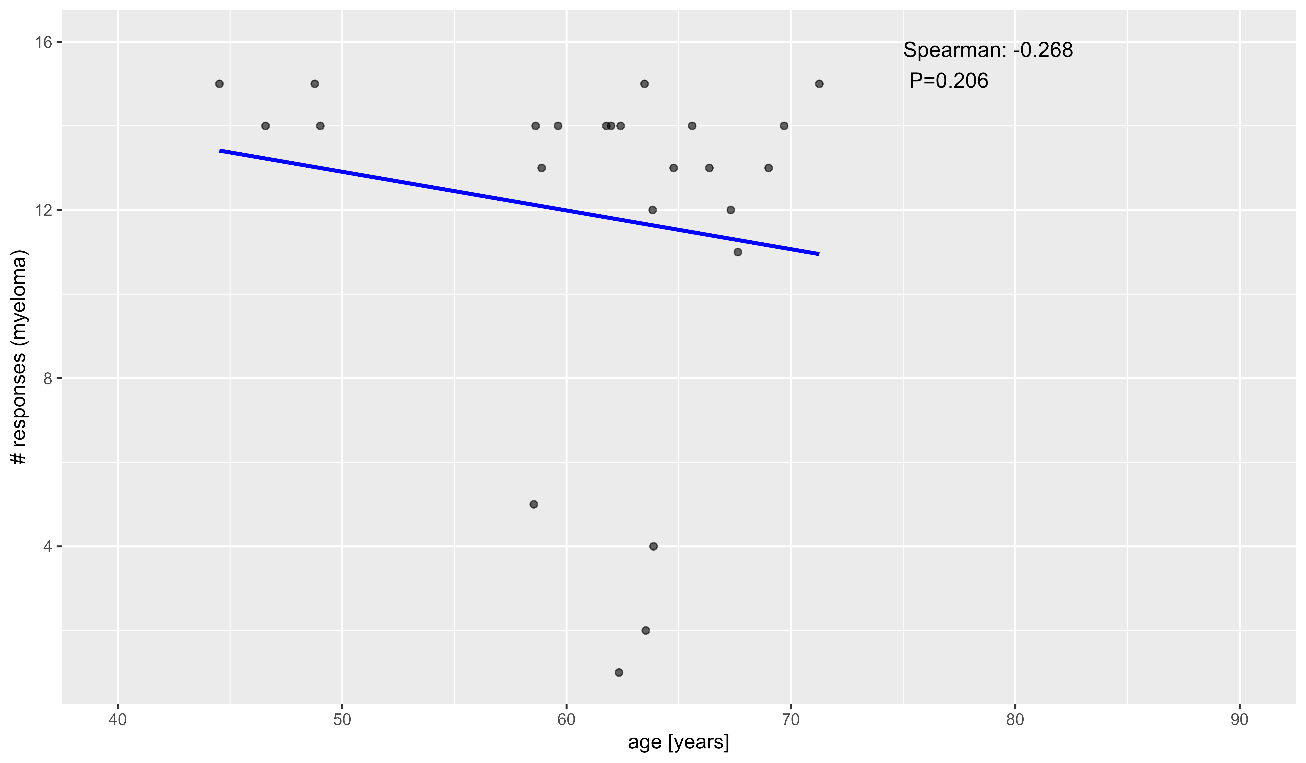


Fig. S12: Scatterplot of responses per patient by age (use case hematology).


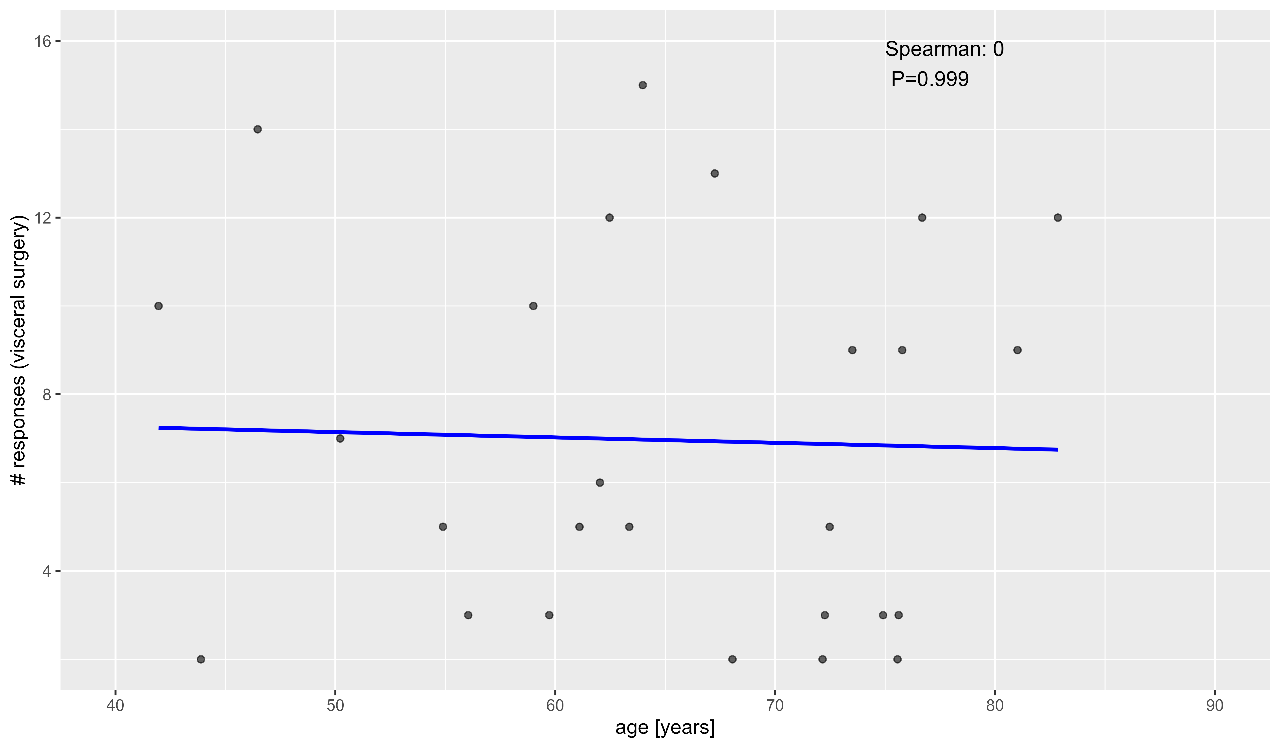


Fig. S13: Scatterplot of responses per patient by age (use case visceral surgery).


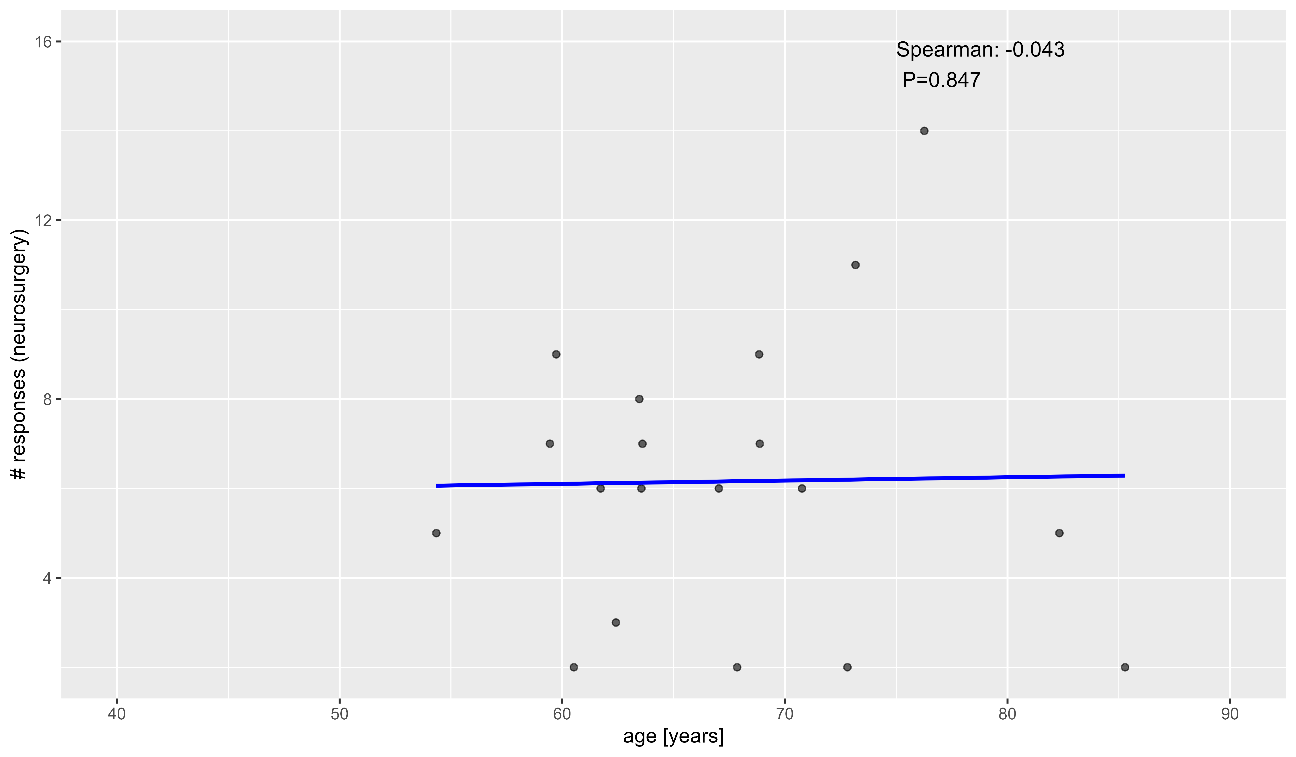


Fig. S14: Scatterplot of responses per patient by age (use case neurosurgery).

## Time from electronic message to patient answer


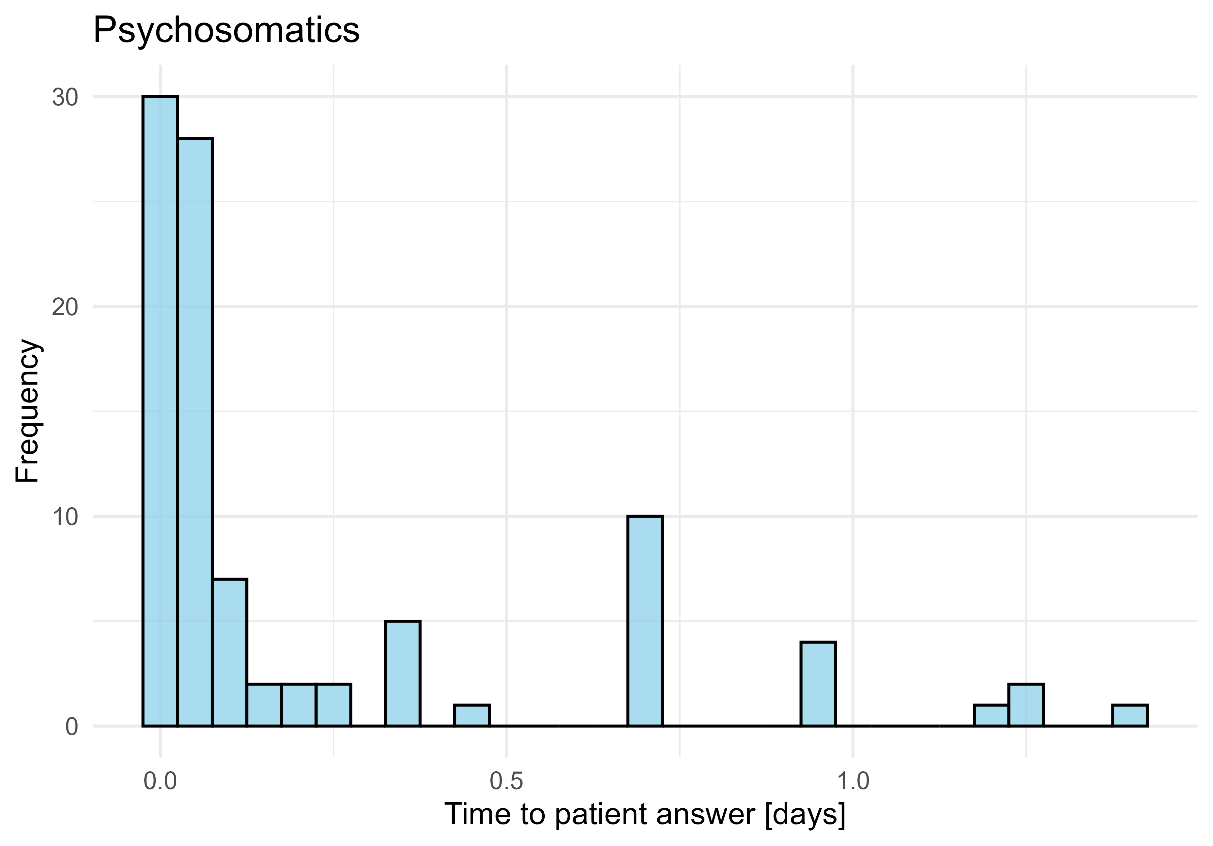


Fig. S15: Histogram of time to patient answer (use case psychosomatics). Median 0.059 days (= 85 min)


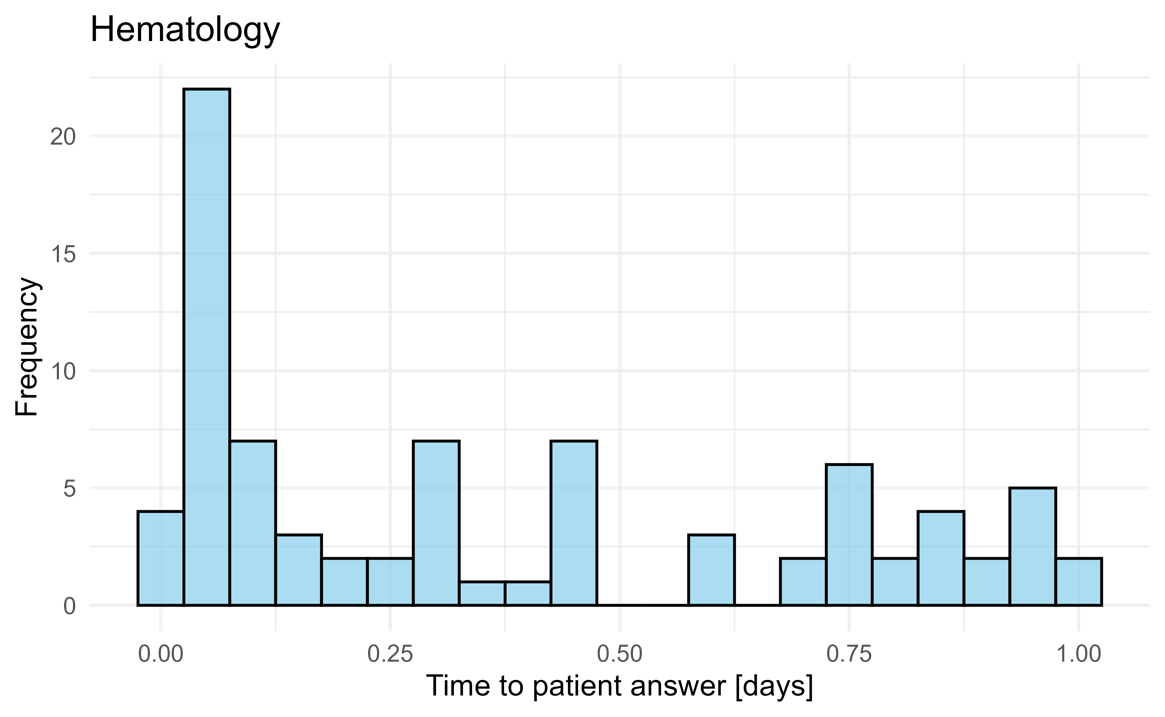


Fig. S16: Histogram of time to patient answer (use case hematology). Median 0.280 days


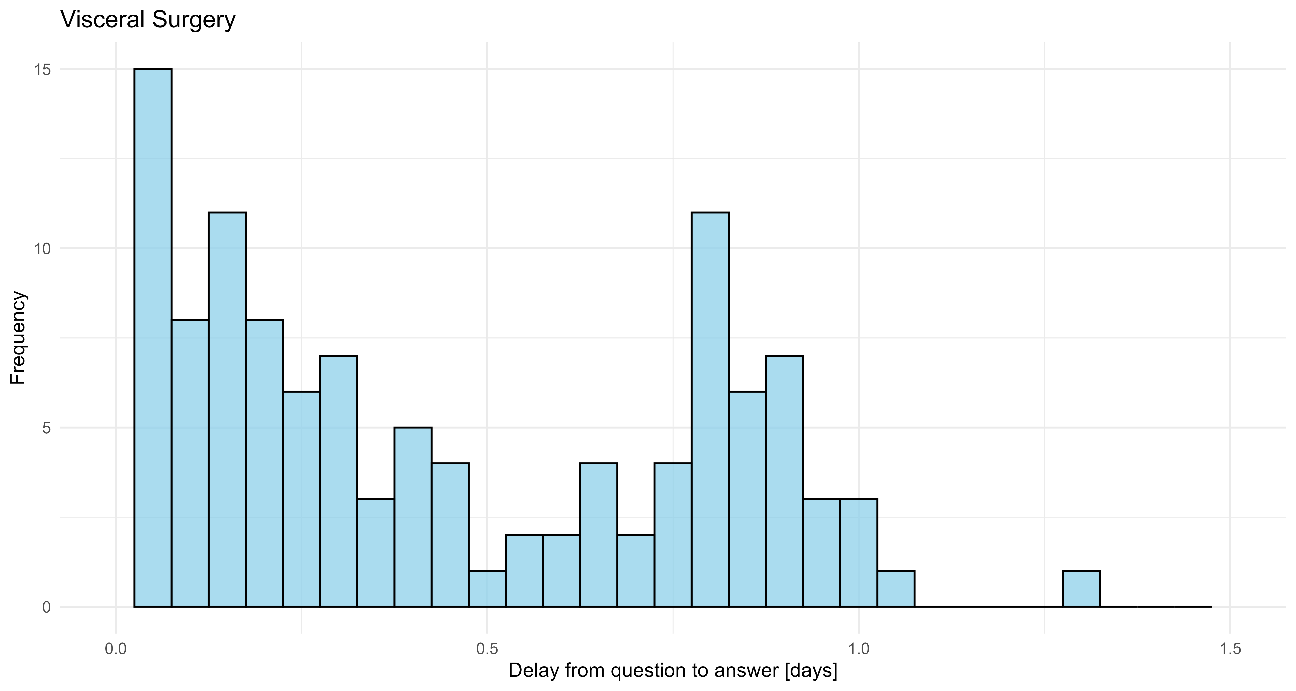


Fig. S17: Histogram of time to patient answer (use case visceral surgery). Median 0.297 days


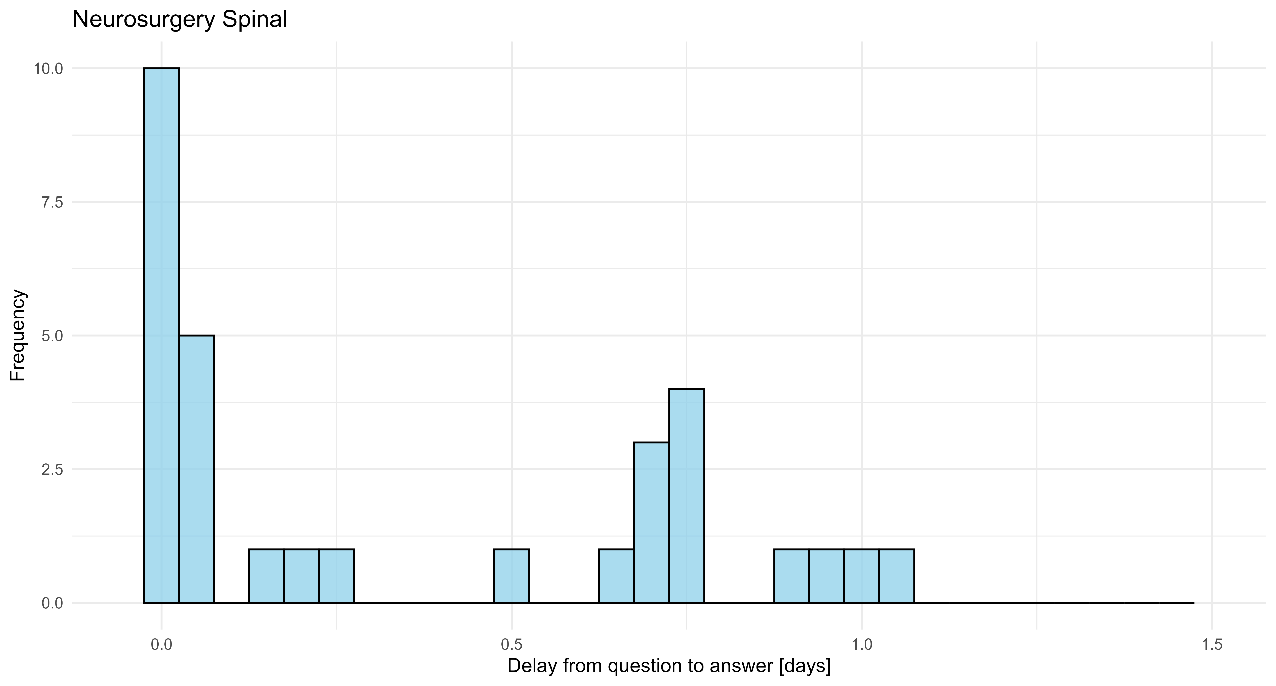


Fig. S18: Histogram of time to patient answer (use case neurosurgery). Median 0.219 days
